# Economic Threshold Analysis of Supplementing Dairy Cow Diets with Betaine and Fat during a Heat Challenge: A Pre- and Post-Experimental Comparison

**DOI:** 10.3390/ani12010092

**Published:** 2021-12-31

**Authors:** Claire D. Lewis, Leah C. Marett, Bill Malcolm, S. Richard O. Williams, Tori C. Milner, Peter J. Moate, Christie K. M. Ho

**Affiliations:** 1 Agriculture Victoria, 1301 Hazeldean Road, Ellinbank, VIC 3821, Australia; claire.lewis@agriculture.vic.gov.au (C.D.L.); leah.marett@agriculture.vic.gov.au (L.C.M.); richard.williams@agriculture.vic.gov.au (S.R.O.W.); tori.c.summers@outlook.com (T.C.M.); peter.moate@agriculture.vic.gov.au (P.J.M.); 2 Centre for Agricultural Innovation, School of Agriculture and Food, Faculty of Veterinary and Agricultural Sciences, The University of Melbourne, Melbourne, VIC 3010, Australia; 3 Faculty of Veterinary and Agricultural Sciences, The University of Melbourne, Parkville, VIC 3010, Australia; b.malcolm@unimelb.edu.au; 4 Agriculture Victoria, 5 Ring Road, Bundoora, VIC 3083, Australia

**Keywords:** heat stress, milk production, canola oil supplement, betaine, economics

## Abstract

**Simple Summary:**

Economic analysis can be used before animal experiments to estimate the change in production that would be needed for experimental treatments to be as equally profitable as the control treatment. After the experiment, the results can be examined to assess if the production threshold was met. This approach was applied before and after an animal experiment that tested the effect of feeding dietary supplements on the milk production of dairy cows experiencing a heat event in climate-controlled chambers. Heat stress reduces the milk yield of cows, but the inclusion of supplements such as betaine and fat could lessen the impact. The pre-experimental economic threshold analysis showed that cows fed a diet containing betaine, fat, or betaine plus fat would need to produce 1%, 9% and 11% more milk, respectively, to be equally as profitable as the control diet. Results from the subsequent animal experiment combined with previously modelled projections of heat stress conditions showed that supplementing diets with fat or betaine, but not in combination could exceed the milk production threshold required to be as profitable as the control diet.

**Abstract:**

Ex ante economic analysis can be used to establish the production threshold for a proposed experimental diet to be as profitable as the control treatment. This study reports (1) a pre-experimental economic analysis to estimate the milk production thresholds for an experiment where dietary supplements were fed to dairy cows experiencing a heat challenge, and (2) comparison of these thresholds to the milk production results of the subsequent animal experiment. The pre-experimental thresholds equated to a 1% increase in milk production for the betaine supplement, 9% increase for the fat supplement, and 11% increase for fat and betaine in combination, to achieve the same contribution to farm profit as the control diet. For the post-experimental comparison, previously modelled climate predictions were used to extrapolate the milk production results from the animal experiment over the annual hot-weather period for the dairying region in northern Victoria, Australia. Supplementing diets with fat or betaine had the potential to produce enough extra milk to exceed the production thresholds, making either supplement a profitable alternative to feeding the control diet during the hot-weather period. Feeding fat and betaine in combination failed to result in the extra milk required to justify the additional cost when compared to the control diet.

## 1. Introduction

Ex ante economic threshold analysis uses the prices of inputs and outputs to estimate the minimum extra benefit required to justify an investment, after allowing for an opportunity cost of capital [1,2]. In agriculture, this approach can be used to estimate the amount of extra output required to make investment in a new management practice economically equivalent to the current practice. For example, Henty and Griffith [3] used this approach to estimate the minimum improvement in milk production needed during heat stress events for an investment in shade infrastructure to return a net present value of 0 Australian Dollar (AUD) at a 10% real discount rate. In dairy nutrition research, the expected size of the production response from an experimental diet compared to the control diet is unknown prior to the experiment being conducted. Economic threshold analysis can be used before an experiment to assess whether the required production response, referred to here as the production threshold, from an experimental diet is likely to be biologically feasible. This information can be used to assess whether the idea in question warrants further investigation, and to enable prioritization and allocation of finite resources to experiments [2].

The objective of this study was to demonstrate how ex ante economic threshold analysis could be applied in a research context, using the example of an experiment where heat stress was induced in dairy cows. Digestion and metabolism of feed generates heat that contributes to the total heat load on dairy cows and when this load cannot be dissipated due to environmental conditions, dry matter intake (DMI) and milk production (milk yield, milk fat and protein yield) are compromised [4,5]. The frequency and severity of heat stress events impacting the dairying regions of Australia are expected to increase in the future [6,7]. The animal experiment was designed to investigate whether supplementing dairy cow diets with betaine and/or fat (as canola oil) could alleviate the negative impacts of a moderate, short-term heat challenge on DMI, milk production and body temperature of the cows, compared to these same parameters in cows fed a control diet [8]. Adding betaine to the diet of animals has been proposed as a method to increase an animal’s resilience to a heat load by potentially reducing cellular damage and reducing the chance of endotoxins escaping the gut moving into the body [9]. This acts to reduce the potential effects of hot weather on the animal and decrease some of the negative impacts on milk production [10,11]. Fat has a lower heat increment than other feeds [12] and feeding high-fat diets to cows during hot weather has been shown to produce more energy-corrected milk than when cows were given lower fat diets [13,14]. While it was hypothesized in the animal experiment that milk production would increase by the dietary inclusion of either betaine supplement and/or additional fat, compared to the control diet, the expected amount of extra milk produced was unknown.

The minimum change in milk production needed for each of the proposed experimental diets to be a profitable alternative to feeding the control diet was estimated by economic threshold analysis during the planning of the heat-stress experiment. Once the animal experiment was completed, the pre-experimental milk production thresholds were compared to the measured differences in milk production from feeding each experimental diet and the control diet. The comparison was made in the context of the Murray Dairy region of northern Victoria and southern New South Wales, Australia. The Murray Dairy region is a major dairy production area, with around 1200 dairy farms producing approximately 20% (1840 million litres) of Australia’s milk [15]. In the region, hot weather periods occur during spring, summer and early autumn, with on average 60 days above 30 °C and 21 days above 35 °C annually, and 74 days where the temperature-humidity index (THI) is 75 or more [7,16]. When THI exceeds 68, cows are likely to experience heat stress [17]. The frequency and severity of extreme temperature events in the Murray Dairy region is expected to increase and further exacerbate production losses in the future [18]. The post-experimental comparison was conducted to examine whether feeding each experimental diet to dairy cows during the annual hot-weather period in northern Victoria could be a profitable alternative to feeding the control diet. The control diet was designed to emulate diets commonly fed on Australian dairy farms during summer.

Our paper reports (1) a pre-experimental economic analysis to estimate the milk production thresholds for an experiment where dietary supplements were fed to dairy cows experiencing a heat challenge, and (2) a comparison of the milk production thresholds to the results of the subsequent animal experiment. The detailed method, results, and in-depth discussion of the animal experiment are reported elsewhere [8].

## 2. Materials and Methods

### 2.1. Pre-Experimental Analysis

The extra costs associated with feeding each experimental diet compared to feeding the control diet alone was used to establish the milk production thresholds. The diets proposed in the animal experiment were formulated as a mixed ration with only the supplement component changing. The three experimental diets were (1) BET—control diet plus 16 g betaine (trimethylglycine as a powder; Feedworks, Romsey, Victoria, Australia) per cow per day, (2) FAT—control diet plus 0.7 kg canola (*Brassica napus* L.) oil per cow per day, or (3) BET + FAT—16 g betaine (trimethylglycine) and control diet plus 0.7 kg canola oil per cow per day. The daily control diet offered to each cow was a total mixed ration (TMR) comprised of 7 kg dry matter (DM) lucerne hay (*Medicago sativa* L.), 6 kg DM pasture silage (predominantly perennial ryegrass, *Lolium perenne* L.), 5.0 kg DM grain mix (500 g/kg wheat grain (*Triticum aestivum* L.), 500 g/kg barley grain (*Hordeum vulgare* L.), 1.5 kg DM solvent extracted canola meal (*Brassica napus* L.), 0.2 kg DM of minerals and vitamins (Ca 134 g/kg, Mg 110 g/kg, P 60 g/kg, Zn 6.4 g/kg, Mn 2.4 g/kg, Cu 1.2 g/kg, I 80 mg/kg, Co 100 mg/kg, Se 24 mg/kg, Vitamin A 165 IU/g, Vitamin D3 24 IU/g, Vitamin E 800 mg/kg), 0.1 kg DM salt, and 42 mL of Bloat Drench (271 g/L alcohols, C12-15 ethoxylated; VicChem, Coolaroo, Victoria, Australia). The chemical compositions of the main dietary components are given in Table 1.

The amount of canola oil fed was restricted to 0.7 kg/cow per day so that the dietary fat concentration did not exceed 70 g/kg DM to avoid a reduction in ruminal fermentation or voluntary DM intake [19]. The betaine dose rate (16 g/cow per day) was the average of dose rates used in experiments from published literature where there had been a positive production response or reduction in heat stress from feeding betaine [10,11,20]. The dose rate also aligned with that recommended by the manufacturer of 15 to 20 g/cow per day [21].

For our pre-experimental economic analysis, all diets were evaluated on a DM offered basis as intake was yet to be measured. Marginal changes in the total DM offered with each diet, such as in conserved fodder or pasture that may be needed to achieve the required changes in milk production, were assumed to come from feed which would otherwise have been grown or fed, but not utilized. Therefore, there were no other costs in addition to the costs of the dietary supplements. It was assumed that the diets were fed every day for 212 days from 1 September, prior to the peak period for hot weather, to 31 March, the end of the hot weather period in northern Victoria. The benefits to milk production from feeding the experimental diets were assumed to be realized within the 212-day feeding period and that no carryover benefits occurred for the remainder of lactation. For each diet, the additional cost of the dietary supplement was calculated assuming an average price of AUD 8.50/kg for betaine and AUD 1.40/kg for vegetable oil, including delivery to northern Victoria, Australia [21,22]. The price of vegetable oil was used instead of canola oil, as this is a more commonly used component of livestock diets, and the two oils were deemed interchangeable as fat supplements. Compared to the control diet, this equated to an extra cost of AUD 0.14/cow per day for the BET diet, AUD 0.98/cow per day for the FAT diet, and AUD 1.12/cow per day for the BET + FAT diet. An 8% p.a. real opportunity cost of variable capital, equating to 4.65% for the 212-day period, was used to calculate the economic threshold (Equation (1)) using an average Victorian real milk (fat + protein) price of AUD 6.28/kg for the period 2013-14 to 2018-19 [23,24]. A litre of milk was assumed to contain 72 g of milk fat plus protein, referred to as milk solids in this analysis. Results were tested using a price sensitivity analysis where the average supplement and milk prices were adjusted by ±20%.

MS_e_ = ($_c_ − $_e_ × (1 + r))/$_MS_ (1)

where MS_e_ equals the extra milk solids required over the 212 days from feeding the experimental diet for it to make an equal contribution to farm profit as the control diet, $_c_ and $_e_ are the costs of the control and the experimental diets, respectively for 212 days, r is the real opportunity cost of variable capital for the 212 days, and $_MS_ is the price per kg of milk solids.

While producers may implement dietary strategies for reasons in addition to immediate milk production responses, such as animal welfare, these factors were not accounted for in the economic analysis presented here.

### 2.2. Animal Heat-Stress Experiment Summary

The animal feeding experiment was conducted in 2018 at Agriculture Victoria Research, Ellinbank (38°14′ S, 145°56′ E), in accordance with the Australian code of practice for the care and use of animals for scientific purposes [25]. The animal experiment is reported in full elsewhere [8] but is described briefly here. Thirty-two multiparous, lactating Holstein-Friesian cows producing 18.6 ± 2.37 kg milk/day (mean ± standard deviation) with 566 ± 47.1 kg bodyweight, 216 ± 18.5 days in milk, 2.7 ± 0.79 parity and 101 ± 2.9 heat tolerance breeding value (DataGene, Bundoora, Victoria, Australia; 100 = national breed mean) were managed in four cohorts of eight cows. Each treatment was assigned at random to two cows within each cohort with treatment groups balanced for bodyweight, milk yield, days in milk and heat tolerance breeding value. Six of the eight cows in each cohort were assigned to six controlled-climate chambers for the heat challenge according to a row-column design. The remaining two cows were retained as spares. Cows had a 7-day covariate period, 14 days adaptation to treatment diets, 3 days of measurement pre-challenge, a 4-day heat challenge where cows were exposed to a moderate heat challenge in controlled-climate calorimeters [26], followed by a 7-day recovery period under ambient conditions.

All cows were offered a common diet during the covariate period comprising 5 kg DM/cow of wheat and 15 kg DM/cow of lucerne hay fed in a paddock per day before being adapted to each treatment diet over a 14-day period. Treatment diets were offered in two equal amounts after the morning and afternoon milkings for 3.5 h each feeding period. Cows were milked twice daily at ~0600 h and ~1500 h and milk yield was measured for each cow at each milking using in-line milk meters. Samples of milk for composition analysis were collected on each day of the covariate period, one day of the pre-heat challenge period (the day closest to thermoneutral conditions) and on each day of the recovery period. During the heat challenge, milk yield was measured by collecting and weighing milk from individual cows at every milking. Milk samples were taken at every milking and milk fat and protein measured using a near infrared milk analyser (model 2000, Bentley Instruments, Chaska, MN, USA). Body temperature was recorded at 15-min intervals during the experiment using intravaginal loggers [27]. Rectal temperature was also measured during the heat challenge at 0600 h and 1500 h.

Temperature-humidity index (THI) was used to define the climate conditions experienced by the cows during the experiment, which replicated those used by Garner et al. [27]. Conditions during the 4-day heat challenge were set to mimic diurnal patterns of previously recorded summer conditions in northern Victoria, with mild heat stress conditions of THI 80 from early morning to midday, increasing to moderate heat stress of THI 84 from midday until evening, and decreasing to mild heat stress conditions of THI 74 overnight.

The results of the animal experiment were subjected to statistical analysis using the following linear model:

$$y\;=\;\mu \;+\;{\beta y}_{\mathrm{cov}}\;+\;F\;+\;B\;+\;\mathrm{FB}\;+\;C\;+\;K\;+\;\varepsilon$$

where y was the outcome variable of interest, y_cov_ was the same variable if available from the covariate period, F was a factor for the effect of added fat, B was a factor for the effect of added betaine and FB their interaction, C was an effect of chamber, K as an effect of cohort and ε a random error for individual animal. The model was applied using ReML software in GenStat 20 (VSN International Ltd., Hemel Hempstead, UK) with fixed effects for the covariate, cohort, chamber, and factorial fat by betaine diet treatments, and with animal as a random effect. Residuals were examined graphically to check distributional assumptions of normality and constant variance.

### 2.3. Post-Experimental Comparison

Results from the animal experiment were combined with the future projections of the mean number of heat stress days (average daily THI greater than 75) predicted from 1 September to 31 March (212 days) for 2025 for the Murray Dairy region by Nidumolu et al. [7,18] to estimate the likely milk solids benefit from feeding each supplement diet for the full feeding period. Across the broader Murray Dairy region, the predicted mean number of consecutive heat stress days for 2025 ranged from three to five days, depending on the severity of the heat challenge [18]. The 4-day heat period of the animal experiment should therefore reasonably represent the type of heat challenges predicted to be experienced in the Murray Dairy region by 2025. Two discrete scenarios were analyzed.

#### 2.3.1. Scenario 1—Benefit Only on Days where THI ≥ 75

The first scenario assumed that feeding each experimental diet would provide a benefit to milk solids production over the control diet only on days when THI was equal to or greater than 75. It was assumed that there were 91 days between 1 September to 31 March where THI was equal to or greater than 75, equal to the 2025 prediction for Tatura (36.4367° S, 145.2328° E) in northern Victoria by Nidumolu et al. [18]. For each of these days, the benefit was calculated as the mean extra milk production (kg milk solids/cow per day) measured during the heat challenge period of the animal experiment, minus the mean extra milk production (kg milk solids/cow per day) during the thermoneutral pre-challenge period of the animal experiment. Subtracting the increase in milk solids production during the pre-challenge period from that during the heat period corrected for the effects of feeding each experimental diet per se. On the remaining 121 days where THI was less than 75, it was assumed the milk solids production from cows fed each experimental diet was the same as that of cows fed the control diet.

#### 2.3.2. Scenario 2—Benefit for All 212 Days

The second scenario aimed to capture all the benefits observed in the experiment of feeding each experimental diet over the control diet. On each of the 91 days where THI was greater than 75, the mean extra milk production (kg milk solids/cow per day) measured during the heat challenge period of the animal experiment was used. On the remaining days where THI was less than 75, each experimental diet was assumed to have a milk production response above the control diet equal to the mean extra milk production (kg milk solids/cow per day) measured during the pre-challenge and recovery periods of the animal experiment.

## 3. Results

### 3.1. Pre-Experimental Analysis

The pre-experimental economic threshold analysis showed that feeding the BET diet over 212 days would need cows to produce 3.2 kg more milk solids per cow to provide the same contribution to farm profit as feeding the control diet (Table 2). This equated to 1 to 2% extra milk solids production for a herd being fed the control diet and averaging 1.7 kg milk solids/cow per day (23 kg milk/cow per day). This required increase was relatively small compared to that resulting from feeding the more expensive FAT diet which required an additional 23.1 to 51.9 kg milk solids/cow (6 to 14% across the herd) over the 212 days for the contribution to farm profit to be the same as the control diet. The combined BET + FAT was the most expensive needing an extra 26.3 to 59.1 kg milk solids/cow to be as profitable as the control diet.

### 3.2. Animal Heat-Stress Experiment Summary

Only the results from the experiment relevant for the economic analysis are presented here. Complete results from the animal experiment are reported in Williams et al. [8]. Milk solids production during the pre-challenge, heat challenge, and recovery periods of the experiment for the different diets are given in Table 3. Statistical differences in milk solids production were only detected by Williams et al. [8] for some treatments. However, the numerical differences between treatments are still useful for an economic analysis and these are reported here. Results from the animal experiment were constrained by the number of animals that completed the full heat challenge period. Raw data within the 3-day pre-challenge period were averaged. Day 2 of the heat challenge was taken to represent the average for the heat event period, a compromise between the heat challenge having an effect on the cows and retaining sufficient animals in the analysis to enable conclusions to be drawn (Williams et al. [8]). The average daily results for the full 7-day recovery period were used.

During the heat challenge and recovery periods of the experiment, all cows fed the three experimental diets increased milk solids production compared to those fed the control diet (Table 3). Cows fed the BET diet produced an additional 0.07 and 0.05 kg milk solids/cow per day (~1.0 and ~0.7 kg milk/cow per day) more than cows fed the control diet during the heat and recovery periods, respectively, with no additional benefit recorded during the pre-challenge period (Table 3). Of the three experimental diets, the FAT diet had the greatest increase in milk solids production in all periods compared to feeding the control diet, with an average daily benefit of 0.31 kg milk solids/cow per day (~4.3 kg milk/cow per day) during the heat challenge (Table 3). The combined BET + FAT diet increased milk solids production in all periods compared to the control diet, with the greatest benefit of 0.11 kg milk solids/cow per day (~1.5 kg milk/cow per day) recorded during the heat challenge period.

### 3.3. Post-Experimental Comparison

#### 3.3.1. Scenario 1—Benefit Only on Days where THI ≥ 75

**Figure 1:**
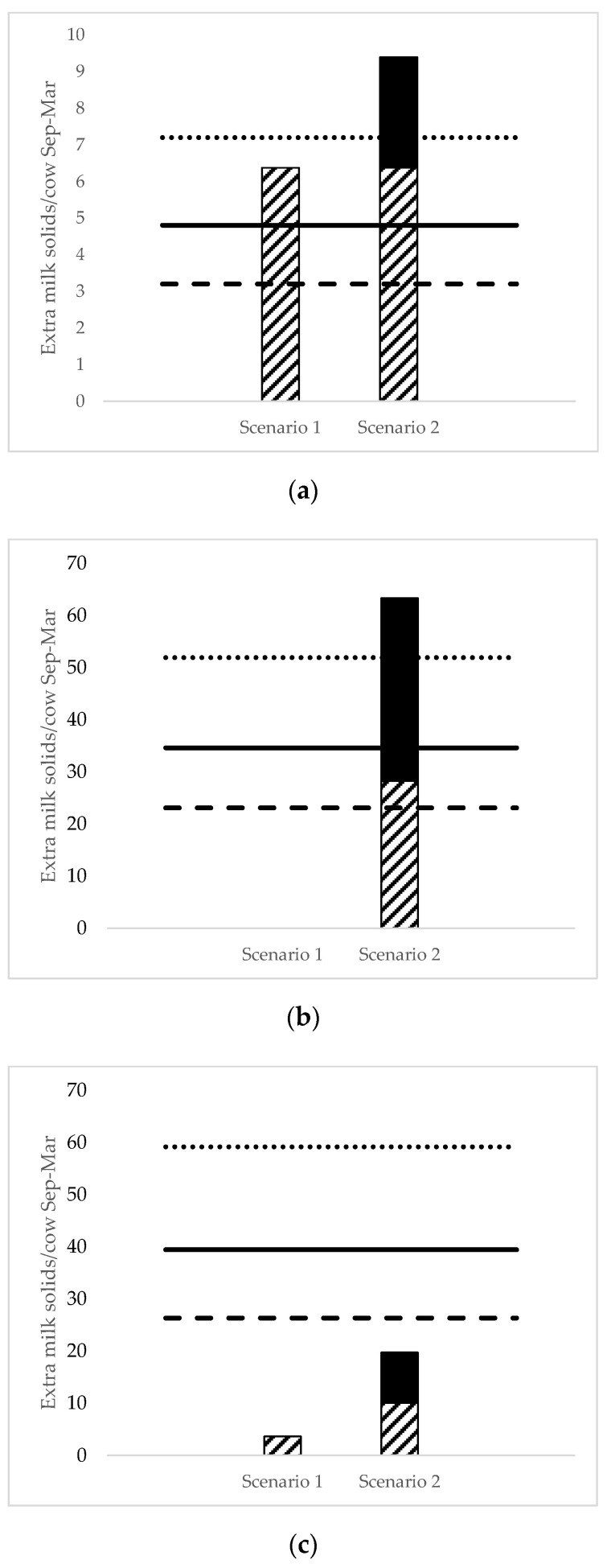
(**a**–**c**) Estimated extra kg milk solids per cow from 1 September to 31 March (212 days) by cows fed (**a**) BET, (**b**) FAT, and (**c**) BET + FAT experimental diets, compared to cows fed the control diet. Two scenarios are presented; Scenario 1 (benefits from feeding each experimental diet were only accounted for when THI ≥ 75), and Scenario 2 (all benefits from feeding each experimental diet were included). Columns represent extra milk solids produced on days where THI ≥ 75 (hatched) and THI < 75 (solid). Horizontal lines represent the required pre-experimental milk production thresholds (extra kg milk solids/cow) for the ‘High supplement price/low milk price’ (dotted line), ‘Average supplement price/average milk price’ (solid line), and ‘Low supplement/high milk price’ (dashed line) combinations.

When benefits during hot weather only were accounted for (Scenario 1), the BET diet was the only diet to be a profitable alternative to the control diet where average supplement and milk prices were assumed. It was estimated that the BET diet would produce an extra 6.4 kg milk solids/cow over the 212-d feeding period compared to the control diet, exceeding the required 4.8 kg milk solids/cow pre-experimental threshold for the diet (Figure 1a). With high supplement price and low milk price conditions, the BET diet was unable to meet the required pre-experimental milk production threshold and was not a profitable alternative to the control diet.

There was no milk production benefit attributed to the FAT diet over the control diet for Scenario 1 (Figure 1b). Feeding the FAT diet produced a similar amount of extra milk solids during the heat challenge period (0.32 kg milk solids/cow per day) as the pre-challenge period (0.31 kg milk solids/cow per day).

For Scenario 1, the BET + FAT diet was estimated to increase milk production by 3.6 kg milk solids/cow compared to the control diet, a much smaller benefit than the required 39.4 kg milk solids/cow threshold with average supplement feed and milk prices (Figure 1c).

#### 3.3.2. Scenario 2—Benefit for All 212 Days

Both the BET diet and the FAT diet were profitable alternatives to feeding the control diet under the assumptions of Scenario 2 (Figure 1a,b). The BET diet was estimated to increase milk production by 9.4 kg milk solids/cow over the 212-d period, with most of the benefit occurring on days where THI ≥ 75 (Figure 1a). Feeding the FAT diet was estimated to produce an extra 63.3 kg milk solids/cow over the 212-d period compared to feeding the control diet, exceeding the required 34.6 kg milk solids/cow under average supplement price and milk price conditions (Figure 1b). Despite the greater milk production benefits in Scenario 2, the BET + FAT diet still failed to be a profitable alternative to the control diet (Figure 1c). The findings of Scenario 2 were consistent across all supplement and milk price combinations tested.

## 4. Discussion

The milk production thresholds estimated in the pre-experimental analysis were biologically feasible, equating to a 1 to 11% increase in milk solids for the experimental diets to be equally as profitable as feeding the control diet. For example, a 7% increase in milk yield has previously been achieved by feeding cows a high fat diet during summer compared to a control diet [13]. After the animal experiment was completed, the ex ante milk production thresholds were compared to the experimental results using scenario analysis. This post-experimental comparison showed that the BET and the FAT diets have potential to be profitable alternatives to the control diet over the hot-weather period experienced in northern Victoria. We acknowledge that numerical, rather than statistically significant, differences in milk production [8] were used in this analysis, and that further research is required to confirm our findings

Despite generating the smallest milk production response of the three experimental diets, feeding betaine had the greatest potential to be a profitable alternative to the control diet. Cows fed the BET diet produced numerically the same amount milk as those fed the control diet during the pre-challenge period but produced on average 0.07 to 0.05 kg milk solids/cow per day more during the heat challenge and recovery periods, respectively. In the post-experimental economic analysis, accounting only for the heat-induced milk production benefits on days where THI ≥ 75 (Scenario 1), the BET diet exceeded the required milk production threshold under average supplement and milk price conditions. If supplement cost was high and milk price was low, the benefits of feeding the BET diet under hot weather conditions alone were not enough to justify the additional cost compared to the control diet. When the benefits recorded during the recovery period of the animal experiment were incorporated into the post-experimental comparison (Scenario 2), the BET diet was a profitable alternative to the control diet under all supplement and milk price conditions tested. The link between the benefits of feeding the BET diet and hot weather conditions means that if conditions vary from those used here for northern Victoria, based on the analysis of Nidumolu et al. [7,18], the profitability of this dietary strategy for managing heat stress will also likely change. This warrants further research under different climatic conditions to understand the industry-wide applicability of betaine as a dietary intervention strategy.

Feeding extra fat alone required cows to produce additional milk solids on each day of the hot weather period to be a profitable alternative to the control diet. Cows fed the FAT diet during the animal experiment produced the greatest amount of milk solids of all treatments. The numerical increase in milk production was not limited to the heat and recovery periods, with a response in milk solids production also recorded during the pre-challenge period, a thermo-neutral period not associated with heat stress. This indicates a benefit for milk production from feeding extra fat per se, irrespective of heat stress conditions and was likely due to higher total energy intake during the pre-challenge period [8]. The post-experimental economic analysis showed that under the 2025 climate conditions predicted for northern Victoria by Nidumolu et al. [7,18], and accounting for the benefits on both hot days (THI ≥ 75) and on days where THI < 75 (Scenario 2), the FAT diet exceeded the required milk production economic thresholds under all price conditions tested. The milk production responses used in the post-experimental comparison of the FAT diet were similar for all days over the hot weather period (extra 0.31 kg milk solids/cow when THI ≥ 75, 0.29 kg milk solids/cow when THI < 75). Practically, this indicates that the benefits of feeding the FAT diet may be insensitive to changes in the frequency of hot days over the September to March period. Further research is required to confirm this result over a longer time than the duration of the animal experiment used here. Dietary supplementation with fat was associated with lower DMI during the heat-challenge period in the animal experiment [8] and this relationship may alter the milk response generated over longer-term exposure to periods of hot weather.

Cows fed betaine and fat in combination did not produce enough extra milk solids to be a profitable alternative to the control diet. This finding held regardless of the scenario used or price conditions tested. During the animal experiment, cows fed the BET + FAT diet exceeded the milk solids produced by cows fed the control diet, but produced less milk solids than those fed the FAT diet. This result was unexpected, and Williams et al. [8] suggest this could be the result of an antagonistic interaction between betaine and canola oil for milk yield. With a combined lower cost and greater milk solids production, feeding the FAT diet was a more profitable option than the BET + FAT diet. However, further research is required to confirm and understand the interaction between the supplements over the longer-term.

The post-experimental analysis used simplified assumptions and the numerical results from a single, short-term experiment to compare to the pre-experimental threshold estimates. A more nuanced approach may yield different conclusions. For example, the cumulative effects of heat stress during a heat challenge on the milk production of dairy cows have previously been reported. Garner et al. [27] showed that the energy corrected milk yield (kg/cow per day) declined by approximately 10% on the first day cows were exposed to a moderate heat challenge, compared to the pre-experimental energy corrected milk yield under ambient conditions. Energy corrected milk yield was reduced by 50% by the fourth day. Accounting for these cumulative effects of heat stress on the milk production of dairy cows as a heat event progresses, as well as the subsequent interaction with dietary intervention strategies, will be important in future analyses. It was also assumed in the post-experimental analysis that the size of the milk solids production benefit was uniform on all days where THI ≥ 75, regardless of the degree of heat stress experienced. However, there are differences in the number of days predicted to induce mild (THI ≥ 75), moderate (THI ≥ 78) and severe (THI ≥ 82) heat stress under future climate conditions [7]. The animal experiment used as a data source in our study was not designed to test the responses of cows offered different diets to different severities of heat stress, and responses may differ depending on the severity of the heat challenge. This is an important area for future work because the profitability of investments to reduce milk production losses from heat stress are influenced by the climatic conditions experienced, with the profitability of an intervention increasing with the severity of heat events [3]. Furthermore, accounting for interactions between dietary interventions and both the length and the severity of heat events will assist in expanding the economic analysis to other dairy regions beyond the northern Victoria example used here. If the full milk production benefits reported here can be achieved in a commercial farm context, dietary supplementation may offer an alternative or complementary strategy to longer-term investments for managing heat stress, such as shade infrastructure [3] and genetic improvement [26,28]. Changes to milk production were the focus of this analysis, but dietary supplementations are likely to impact ruminants in other ways, with changes in body temperature and dry matter intake also reported [8,12,20,29,30]. These additional considerations may have important, longer-term implications for animal production, welfare and profitability which should be accounted for in individual decision making.

## 5. Conclusions

Pre-experimental economic threshold analysis can be used in research prior to an experiment to identify the minimum production responses required for an experimental treatment to be a profitable alternative to the control treatment. The biological feasibility of achieving the production threshold can then help with experimental design and resource allocation. Comparing the estimated pre-experimental production thresholds to the results of the subsequent experiment can then provide a useful insight into the likelihood of achieving the required production benefits in a commercial farm context.

## Figures and Tables

**Table 1:** Composition of the main dietary ingredients used in the animal experiment (as published by Williams et al. 2021).

Parameter	Grain Mix ^1^	Lucerne Hay	Pasture Silage
Crude protein (g/kg DM)	194	168	176
Neutral detergent fibre (g/kg DM)	153	457	508
Starch (g/kg DM)	486	16	5
Crude fat (g/kg DM)	27.5	18.6	51.4
Metabolisable energy (MJ/kg DM)	13.7	9.4	10.5

^1^ Grain mix consisted of 500 g/kg wheat grain and 500 g/kg barley grain.

**Table 2:** Pre-experimental economic threshold analysis. Extra diet cost (AUD/cow), and extra milk required (kg milk solids/cow) over 212 days for the BET, FAT, and BET + FAT diets to be equally as profitable as the control diet, at a range of feed and milk prices and allowing for an 8% p.a. real opportunity cost. Per day figures are given in parentheses. The average percentage change in production was calculated assuming cows averaged 1.7 kg milk solids/day (23 kg milk/day) for 212 days when fed the control diet.

vs. Control	Extra Diet Cost (AUD/Cow)	Extra Milk Required (kg Milk Solids/Cow)	Extra Milk Required (kg/Cow)	Extra Milk Required (%)
High supplement price/Low milk price				
BET	36 (0.17)	7.2 (0.03)	99 (0.47)	2%
FAT	261 (1.23)	51.9 (0.24)	711 (3.35)	14%
BET + FAT	297 (1.40)	59.1 (0.28)	810 (3.82)	16%
Average supplement price/Average milk price				
BET	30 (0.14)	4.8 (0.02)	66 (0.31)	1%
FAT	217 (1.02)	34.6 (0.16)	474 (2.24)	9%
BET + FAT	248 (1.17)	39.4 (0.19)	540 (2.55)	11%
Low supplement price/High milk price				
BET	24 (0.11)	3.2 (0.02)	44 (0.21)	<1%
FAT	174 (0.82)	23.1 (0.11)	316 (1.49)	6%
BET + FAT	198 (0.93)	26.3 (0.12)	360 (1.70)	7%

**Table 3:** Daily mean milk solids production (kg milk solids/cow per day) of cows fed the control, BET, FAT and BET + FAT diets for the pre-challenge, heat challenge and the recovery periods of the experiment. Numerical differences between the control diet and each experimental diet are shown in parentheses.

	Pre-Challenge Period (kg MS/Cow per Day)	Heat Challenge Period (kg MS/Cow per Day)	Recovery Period (kg MS/Cow per Day)
Control	1.63	1.50	1.45
BET	1.63 (0.00)	1.57 (0.07)	1.51 (0.05)
FAT	1.95 (0.32)	1.81 (0.31)	1.71 (0.26)
BET + FAT	1.69 (0.07)	1.61 (0.11)	1.55 (0.09)

## Data Availability

Data used in this study have been referenced in the manuscript.
